# The Role of Serum Uric Acid in the Prediction of Type 2 Diabetes Mellitus: Tehran Lipid and Glucose Study

**DOI:** 10.1002/jcla.70314

**Published:** 2026-07-24

**Authors:** Nahid Kord, Samaneh Asgari, Maryam Tohidi, Somayeh Hosseinpour‐Niazi, Fereidoun Azizi, Farzad Hadaegh

**Affiliations:** ^1^ Prevention of Metabolic Disorders Research Center, Research Institute for Metabolic and Obesity Disorders, Research Institute for Endocrine Sciences Shahid Beheshti University of Medical Sciences Tehran Iran; ^2^ Nutrition and Endocrine Research Center, Research Institute for Endocrine Disorders, Research Institute for Endocrine Sciences Shahid Beheshti University of Medical Sciences Tehran Iran; ^3^ Endocrine Research Center, Research Institute for Endocrine Disorders, Research Institute for Endocrine Sciences Shahid Beheshti University of Medical Sciences Tehran Iran

**Keywords:** incident, prediction model, serum uric acid, Tehran Lipid and Glucose Study, type 2 diabetes mellitus

## Abstract

**Background:**

To investigate the association between serum uric acid (SUA) and incident type 2 diabetes mellitus (T2DM) and its potential role in improving the well‐known predictive models of disease.

**Methods:**

The study included 5719 subjects (2588 men) aged ≥ 30 years without T2DM at baseline. Multivariate Cox proportional hazard analyses were applied using SUA as continuous and categorical variables. The added value of SUA was examined by applying Harrell's concordance statistic (C‐index) and integrated discrimination improvement (IDI).

**Results:**

During a median follow‐up of 8.2 years, 778 (376 men) incident T2DM occurred. Each 1‐mg/dL increment in SUA was associated with a higher risk of disease in the whole population, men, and women (HR [95% CI]: 1.18 [1.11–1.26], 1.19 [1.09–1.31], and 1.26 [1.14–1.38], respectively). This association in the whole population persisted after adjustment for homeostasis model assessment of insulin resistance and nutritional data (1.15 [1.06–1.25] and 1.19 [1.10–1.30], respectively). A rising risk of incident T2DM was present across quartiles (Q1: reference) in the whole population, reaching 1.82 (1.39–2.37) for Q4 (*p*‐trends < 0.001). Adding SUA to the well‐known models did not significantly improve the risk prediction.

**Conclusion:**

Although SUA has a significant linear association with incident T2DM, its addition to the well‐known models will not be accompanied by better risk prediction.

## Graphical abstract

This study aimed to examine the association between serum uric acid and incident type 2 diabetes mellitus. The study included 5719 subjects (2588 men) aged ≥ 30 years without T2DM at baseline. Multivariate Cox proportional hazard analyses were applied using SUA as continuous and categorical variables. Subjects followed up during a median follow‐up of 8.2 years. Each 1‐mg/dL increment in SUA was associated with a higher risk of incident T2DM in the whole population, men, and women (hazard ratio [HR] and 95% confidence interval [CI]: 1.18 [1.11–1.26], 1.19 [1.09–1.31], and 1.26 [1.14–1.38], respectively); a relationship that was not affected by sex, age categories, and obesity status (all *p*s for interaction > 0.59). Considering SUA as a categorical variable, a rising risk of incident T2DM was present across quartiles (Qs) (Q1: reference) in the whole population, reaching 1.82 (1.39–2.37) for Q4 (*p* for trends < 0.001). Similarly, increasing values of SUA were accompanied by incident T2DM in both sexes (*p*s for trend ≤ 0.002). The association between SUA and incident T2DM persisted after further adjustment for homeostasis model assessment of insulin resistance (HOMA‐IR) and nutritional data; the corresponding HR (95% CI) for each 1‐mg/dL increase of SUA in the whole population was 1.15 (1.06–1.25) and 1.19 (1.10–1.30), respectively.

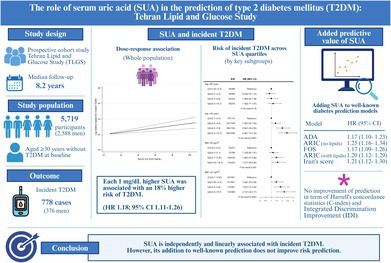

**Abbreviations**
2 h‐PCG: 2‐h post‐challenge glucose
ADA: American Diabetes Association
ARIC: Atherosclerosis Risk in Communities study
BMI: body mass index
CI: confidence interval
C‐index: concordance statistic
CKD‐EPI: chronic kidney disease epidemiology collaboration
Cr: creatinine
CV: coefficient of variation
CVD: cardiovascular disease
DALY: disability‐adjusted life‐years
DBP: diastolic blood pressure
eGFR: estimate glomerular filtration rate
FH‐DM: family history of diabetes
FOS: Framingham Offspring Study
FPG: fasting plasma glucose
GLUT9: glucose transporter 9
GSIS: glucose‐stimulated insulin secretion
HDL‐C: high‐density lipoprotein cholesterol
HOMA‐IR: homeostasis model assessment of insulin resistance
HR: hazard ratios
IDF: International Diabetes Federation
IDI: Integrated Discrimination Improvement
iNOS: inducible nitric oxide synthase
MAQ: Modifiable Activity Questionnaire
MENA: Middle East and North Africa
MET: metabolic equivalent of task
NCDs: non‐communicable diseases
NO: nitric oxide
SBP: systolic blood pressure
SD: standard deviation
SSBs: sugar‐sweetened beverages
SUA: serum uric acid
T2DM: type 2 diabetes mellitus
TG: triglycerides
TLGS: Tehran Lipid and Glucose Study
WC: waist circumference

## 1 Introduction

Type 2 diabetes mellitus (T2DM) is a growing disease worldwide, which imposes life‐threatening, disabling, and costly complications and reduces life expectancy. In 2021, the global diabetes prevalence was estimated to be 10.5% (536.6 million people) and rise to 12.2% (783.2 million) in 2045 [1]. Furthermore, in 2019, the global age‐standardized disability‐adjusted life‐years (DALY) and death rates were 801.5 and 18.5 per 100,000 with an increase of 27.6% and 10.8% since 1990, respectively [2].

According to the 11th edition of the International Diabetes Federation (IDF) report, the Middle East and North Africa (MENA) ranked as the first region with the highest prevalence of T2DM in people aged 20–79 years in 2024 (i.e., 19.9%) [3]. Furthermore, Iran, with about 5.5 million person with diabetes ranked third among MENA countries in term of the number of adults with diabetes [3]. The annual incidence of T2DM in the Iranian population was reported to be over 1% [4].

Along with known risk factors for T2DM, serum uric acid (SUA), as the ultimate product of purine metabolism, has been proposed as a risk factor in the development of T2DM through different mechanisms [5, 6, 7]. The results of a meta‐analysis among cohort studies indicated that each 1 mg/dL increment in SUA led to a 13.1% increase in the risk of T2DM, with a high heterogeneity between included studies (*I* ^2^ = 51.9%) [8]. Moreover, previous studies have reported inconsistent patterns in the association between SUA and incident T2DM, including linear, L‐shaped, and U‐shaped relationships [9, 10, 11, 12, 13, 14, 15, 16, 17].

In the current study, we intend to investigate the long‐term association between SUA and incident T2DM and its potential role for improving the prediction of disease in a large population‐based study called Tehran Lipid and Glucose Study (TLGS).

## 2 Material and Methods

TLGS is a community‐based, prospective cohort study involving an urban population in Tehran aged 3 years and older. It was initiated primarily to identify the prevalence and incidence of non‐communicable diseases (NCDs) and associated risk factors. Participant recruitment occurred in two stages: the first phase from 1999 to 2002 with 15,005 individuals, and the second phase from 2002 to 2005 with 3550 individuals. The study planned to conduct follow‐up over a minimum of 20 years at three‐year intervals (i.e., third phase: 2005 to 2008, fourth phase: 2009 to 2011, fifth phase: 2012 to 2015, sixth phase: 2015 to 2018, and seventh phase: 2018 to 2022). Detailed information on the design and methodology of the TLGS has been published previously [18].

### 2.1 Study Population

Of the 8510 total population aged ≥ 30 years who participated in the 4th phase of TLGS, individuals with a history of anti‐diabetic medications until phase 4 (*n* = 855), or prevalent T2DM at phase 4 (*n* = 524) were excluded, leaving 7131 individuals. After excluding subjects with missing data on covariates (*n* = 998) and those with no follow‐up (*n* = 414), 5719 participants remained.

The Institutional Review Board of the Research Institute for Endocrine Sciences, Shahid Beheshti University of Medical Sciences, Tehran, Iran, approved this study. From all participants, a written informed consent was obtained.

### 2.2 Clinical and Laboratory Measurements

Data on demographics, history of diseases and medications, family history of NCDs, and smoking habits were collected through a questionnaire by trained physicians. To assess physical activity, including all three types of activity that are, leisure time, job, and household activities in the past year, the Modifiable Activity Questionnaire (MAQ) was applied.

Anthropometric parameters including weight, height, and waist circumference (WC) were measured with light clothing and without shoes in a standing position and shoulders in normal alignment, using a digital scale (Seca 707, Seca Corp; range 0.1–150 kg, sensitivity 0.1 kg), and a tape meter. WC was measured at the umbilical level. Body mass index (BMI) was calculated as weight (kg) divided into the square of height (m^2^). To determine the systolic and diastolic blood pressures (SBP and DBP, respectively), subjects rested for 15 min, then two measurements were performed using a standardized mercury sphygmomanometer (calibrated by the Iranian Institute of Standards and Industrial Research) on the right arm, and blood pressure was considered as the mean of these measurements.

After 12–14 h of overnight fasting, a blood sample was taken from each participant. For all participants without diabetes, a standard oral glucose tolerance test with 82.5‐g monohydrate glucose (equivalent to 75 g anhydrous glucose) was performed. All biochemistry analyses including fasting plasma glucose (FPG), 2‐h post‐challenge glucose (2 h‐PCG), triglycerides (TG), high‐density lipoprotein cholesterol (HDL‐C), creatinine (Cr), and SUA were done in the TLGS research laboratory using a Selectra 2 biochemistry analyzer (Vital Scientific, Spankeren, The Netherlands). Blood glucose and lipid parameters were assayed by direct enzymatic colorimetric methods, and serum creatinine measurement was done using the kinetic colorimetric Jaffe method by commercial kits (Pars Azmoon Inc., Iran) on the same day of sampling. For determination of SUA level, an enzymatic photometric method using Uricase and TOOS (N‐ethyl‐N‐(hydroxy‐3‐sulfopropyl)‐m‐toluidin) with commercial kits (Zist‐chimi, Iran) was used in the stored serum samples at −80°C. Monitoring of the quality of assays was applied using commercial serum controls in two different concentrations. Both intra‐ and inter‐assay coefficients of variation (CVs) for glucose, Cr, and lipid measures were less than 3.0%, and the corresponding values for SUA were less than 2.7%. For the measurement of serum insulin, electrochemiluminescence immunoassay method with the Roche Diagnostics kits and a Roche/Hitachi Cobas e‐411 analyzer (GmbH, Manheim, Germany) was used; the related intra‐ and inter‐assay CVs of 1.2% AND 3.5%, respectively.

### 2.3 Variable Definitions

In the current study, T2DM was defined as having FPG ≥ 126 mg/dL and/or 2 h‐PCG ≥ 200 mg/dL or the use of anti‐diabetic medications. The ratio of TG to HDL‐C was calculated by dividing the values of TG by HDL‐C. To estimate glomerular filtration rate (eGFR), the chronic kidney disease epidemiology collaboration (CKD‐EPI) formula was used [19]. Homeostasis model assessment of insulin resistance (HOMA‐IR) was calculated as (fasting insulin [μU/mL] × fasting glucose [mmol/L])/22.5 [20].

Educational levels were categorized into three groups: < 6, ≥ 6 and < 12, and ≥ 12 years of formal education. Low physical activity was defined as the metabolic equivalent of task (MET) < 600 min/week. Current smokers were defined as those who used any tobacco product (cigarette, pipe, and water pipe) at the time of examination. Prevalent cardiovascular disease (CVD) was defined as a positive history of acute coronary syndrome leading to admission, history of percutaneous coronary intervention, coronary artery bypass graft, angiographic proven coronary artery disease, or history of stroke. The family history of diabetes (FH‐DM) was defined as T2DM in any first‐degree relatives. Due to the lack of consistently available detailed information on menstrual history for all participants, and based on previous studies reporting the average age at natural menopause among Iranian women, women aged ≥ 50 years were considered postmenopausal [21, 22].

### 2.4 Statistical Analysis

Baseline characteristics of the study population were described as mean ± standard deviation (SD) for continuous variables and frequencies (%) for categorical variables by SUA quartiles. Quartiles were determined for the total population and separately for each gender. The baseline characteristics of the participants across quartiles of SUA were compared using the ANOVA test for normally distributed continuous variables, the Chi‐squared test for categorical variables, and the Kruskal–Walli's test for skewed (i.e., TG) and ordered variables.

To be able to capture a potential nonlinear association between the SUA and incident T2DM, multivariable restricted cubic splines with 4 knots, which defined the 5th, 25th, 75th, and 95th percentiles, were used [23]. To evaluate the association of SUA quartiles and 1‐mg/dL increase of SUA with the incident T2DM, Cox proportional hazard models were applied; model 1 was adjusted for age and sex; model 2 was further adjusted for BMI, WC, education, current smoking, low physical activity, history of CVD, FH‐DM, anti‐hypertensive medications, lipid‐lowering medications, SBP, TG/HDL‐C, eGFR, and FPG (as referenced in the results section as the fully adjusted model). Moreover, in the sex‐stratified analysis among women, we added menopausal status as another covariate. The adjusted hazard ratios (HRs) and 95% confidence intervals (CIs) were reported for each of the SUA quartiles, considering the first quartile as a reference category. As sensitivity analyses, first, we examined the effect modification of obesity status and age categories on the association between SUA and incident T2DM. Second, we also considered the potential confounding effect of HOMA‐IR in place of FPG in multivariable analysis. Third, we also considered the potential dietary confounder of total energy intake, red meat, organ meat, dairy products, and sugar‐sweetened beverages (SSBs) in subpopulations with the relevant data.

The event date for incident T2DM was described as the mid‐time between the date of the follow‐up visit at which outcome was detected for the first time and the most recent follow‐up visit prior to the diagnosis; the follow‐up time was drawn from the difference between the calculated mid‐time date and the date at which the subjects entered the study. For the censored and lost to follow‐up individuals, the survival time was between the first and the last observation dates. The proportionality in the Cox models was evaluated with the Schoenfeld residual test; generally, all proportionality assumptions were appropriate. Although there was no sex interaction (*p*‐value for interaction = 0.99), to be comparable with other studies, all analyses were done in the total population as well as in men and women separately.

We investigated the potential role of SUA in improving the prediction of incident T2DM by applying five predictive models which were previously validated for the Iranian population [24, 25], including the American Diabetes Association (ADA) (non‐invasive model) [26], the Atherosclerosis Risk in Communities study (ARIC) (with and without lipid measures) [27], the Framingham Offspring Study (FOS) [28], as well as the Iranian Risk Score [29]. Harrell's concordance statistic (C‐index) was calculated to assess the discriminative power of the models. We further applied Integrated Discrimination Improvement (IDI) to evaluate the predictive value of SUA for incident T2DM [30]. Bias‐corrected 95% CIs were delivered using the bootstrapping method with 1000 replications [30, 31].

All analyses were conducted using STATA version 17 SE (Stata Corp, TX, USA), and a two‐tailed *p* < 0.05 was considered significant.

## 3 Results

The study population consisted of 5719 participants (2588 men) with a mean age (SD) of 48.02 (12.42) years. The baseline characteristics of the study participants stratified by quartiles of SUA for the whole population are presented in Table 1. Compared to the participants in the first quartile, those in the fourth quartile were older. They had higher values of BMI, WC, SBP, DBP, FPG, TG, and SUA, and lower values of HDL‐C and eGFR. Furthermore, those in the fourth quartile had more frequency of current smoking, a positive history of CVD, and lower physical activity as well as using anti‐hypertensive and lipid‐lowering medications. Regarding the education level, significant differences were present among quartiles. The baseline characteristics of men and women are shown in Supplementary Tables 1 and 2, respectively.

**TABLE 1:** Baseline characteristics of the study participants by SUA quartiles: Tehran Lipid and Glucose Study (2009–2012).

Variables*	Whole population *n* = 5719	SUA (mg/dL) quartiles				
		Q1 (≥ 0.7 to < 4.05) *n* = 1497	Q2 (≥ 4.05 to < 5.00) *n* = 1511	Q3 (≥ 5.00 to < 5.85) *n* = 1316	Q4 (≥ 5.85 to < 10.70) *n* = 1395	*p* **
Age (years)	48.02 ± 12.42	45.14 ± 11.26	48.80 ± 12.40	49.50 ± 12.72	48.88 ± 12.87	< 0.001
Sex (men, %)	2588 (45.25)	147 (9.82)	481 (31.83)	810 (61.55)	1150 (82.44)	< 0.001
WC (cm)	95.15 ± 11.22	89.82 ± 10.58	94.13 ± 10.91	97.31 ± 10.67	99.94 ± 10.02	< 0.001
BMI (kg/m^2^)	28.32 ± 8.73	27.17 ± 4.33	28.29 ± 4.82	28.97 ± 16.12	28.99 ± 4.48	< 0.001
SBP (mmHg)	116.62 ± 17.33	110.76 ± 15.97	115.75 ± 16.89	118.91 ± 17.55	121.67 ± 17.05	< 0.001
DBP (mmHg)	77.69 ± 10.75	73.91 ± 10.06	77.07 ± 10.22	79.08 ± 10.50	81.10 ± 10.90	< 0.001
FPG (mg/dL)	94.32 ± 8.98	91.91 ± 8.18	94.00 ± 8.76	95.60 ± 9.35	96.06 ± 9.08	< 0.001
TG (mg/dL)	127 (90–178)	98 (72–133)	122 (88–168)	137.5 (101–186)	165 (119–228)	0.001
HDL‐C (mg/dL)	47.26 ± 11.34	52.31 ± 11.35	48.83 ± 11.29	45.58 ± 10.30	41.73 ± 9.36	< 0.001
SUA (mg/dL)	4.97 ± 1.33	3.41 ± 0.48	4.51 ± 0.26	5.38 ± 0.26	6.76 ± 0.79	< 0.001
eGFR (mL/min/1.73m^2^)	81.81 ± 13.52	84.66 ± 12.64	81.58 ± 13.15	81.29 ± 13.21	79.48 ± 14.56	< 0.001
Current smoker (yes, %)	1077 (18.83)	155 (10.35)	237 (15.68)	293 (22.26)	392 (28.10)	< 0.001
Low physical activity (yes, %)	2263 (39.57)	572 (38.21)	552 (36.53)	541 (41.11)	598 (42.87)	0.002
Education (*n*, %)						
< 6 years	1286 (22.49)	308 (20.57)	404 (26.74)	304 (23.10)	270 (19.35)	< 0.001
6–12 years	3101 (54.22)	840 (56.11)	805 (53.28)	730 (55.47)	726 (52.04)	
> 12 years	1332 (23.29)	349 (23.31)	302 (19.99)	282 (21.43)	399 (28.60)	
History of CVD (yes, %)	395 (6.91)	52 (3.47)	105 (6.95)	116 (8.81)	122 (8.75)	< 0.001
FH‐DM (yes, %)	548 (9.58)	159 (10.62)	150 (9.93)	114 (8.66)	125 (8.96)	0.264
Lipid‐lowering medications (yes, %)	381 (6.66)	56 (3.74)	120 (7.94)	99 (7.52)	106 (7.60)	< 0.001
Antihypertensive medications (yes, %)	558 (9.76)	84 (5.61)	160 (10.59)	141 (10.71)	173 (12.40)	< 0.001

Abbreviations: BMI, body mass index; CVD, cardiovascular diseases; DBP, diastolic blood pressure; eGFR, estimated glomerular filtration rate; FH‐DM, family history of type 2 diabetes mellitus; FPG, fasting plasma glucose; HDL‐C, high‐density lipoprotein cholesterol; IQR, interquartile range; *n*, number; Q, quartile; SBP, systolic blood pressure; SD, standard deviation; SUA, serum uric acid; TG, triglycerides; WC, waist circumference.

* Data are presented as mean ± SD for normally distributed continuous variables, median (IQR) for triglycerides (TG) with a skewed distribution, and number (%) for categorical variables.

** *p*‐values correspond to the ANOVA test for normally distributed continuous variables, the Chi‐squared test for categorical variables, and the Kruskal–Wallis test for skewed or ordinal variables.

During a median follow‐up of 8.2 years (interquartile range: 4.7–9.7), 778 incident T2DM (376 men) were identified. The annual crude incidence rate of T2DM per 1000 person‐year (95% CI) was 18.4 (17.1–19.7), 20 (18.0–22.2), and 17.1 (15.5–18.9) in the whole population, men and women, respectively. The incidence rate (95% CI) of T2DM by quartiles of SUA is presented in Table 2.

**TABLE 2:** HRs (95% CIs) for incident T2DM by quartiles and 1 mg/dL increase of SUA.

Whole population	Quartiles of SUA (mg/dL)*					1 mg/dL increase of SUA
	Q1 (≥ 0.7 to < 4.05)	Q2 (≥ 4.05 to < 5.00)	Q3 (≥ 5.00 to < 5.85)	Q4 (≥ 5.85 to < 10.70)	*p* for trend	
*E*/*N*	117/1497	177/1511	219/1316	265/1395		—
Incidence rate**	9.6 (8.0–11.5)	15.7 (13.6–18.2)	23.5 (20.6–26.9)	27.8 (24.7–31.4)		—
Model 1	Reference	1.58 (1.25–2.01)	2.54 (2.00–3.23)	3.25 (2.54–4.16)	< 0.001	1.36 (1.29–1.44)
Model 2	Reference	1.28 (1.01–1.63)	1.57 (1.23–2.02)	1.82 (1.39–2.37)	< 0.001	1.18 (1.11–1.26)
Men	Q1 (≥ 2.15 to < 4.05)	Q2 (≥ 4.05 to < 5.00)	Q3 (≥ 5.00 to < 5.90)	Q4 (≥ 5.90 to < 10.30)	*p* for trend	
*E*/*N*	8/147	49/481	116/810	203/1150		—
Incidence rate**	6.8 (3.4–13.5)	13.5 (10.2–17.8)	19.7 (16.4–23.6)	25.2 (22.0–28.9)		—
Model 1	Reference	2.09 (0.99–4.42)	3.11 (1.52–6.38)	4.21 (2.08–8.54)	< 0.001	1.33 (1.23–1.45)
Model 2	Reference	1.74 (0.82–3.68)	1.99 (0.96–4.10)	2.47 (1.20–5.06)	0.002	1.19 (1.09–1.31)
Women	Q1 (≥ 0.70 to < 4.10)	Q2 (≥ 4.10 to < 5.00)	Q3 (≥ 5.00 to < 5.85)	Q4 (≥ 5.85 to < 10.70)	*p* for trend	
*E*/*N*	109/1350	128/1030	103/506	62/245		—
Incidence rate**	9.9 (8.2–11.9)	16.8 (14.2–20.0)	30.3 (24.9–36.7)	42.4 (33.0–54.4)		—
Model 1	Reference	1.57 (1.21–2.03)	2.68 (2.04–3.53)	3.28 (2.37–4.54)	< 0.001	1.44 (1.32–1.55)
Model 2	Reference	1.31 (1.01–1.72)	1.71 (1.28–2.30)	2.03 (1.42–2.91)	< 0.001	1.26 (1.14–1.38)

*Note:* Model 1: adjusted for age and sex in the whole population, and just for age in the sex‐stratified analyses. Model 2 (full adjustment model): Model 1 + adjusted for BMI, WC, education, current smoker, low physical activity, history of CVD, FH‐DM, anti‐hypertensive medications, lipid‐lowering medication, SBP, TG/HDL‐C, eGFR, and FPG (+ menopausal status among women).

Abbreviations: BMI, body mass index; CI, confidence interval; CVD, cardiovascular diseases; *E*, number of events; eGFR, estimated glomerular filtration rate; FH‐DM, family history of type 2 diabetes mellitus; FPG, fasting plasma glucose; HDL‐C, high‐density lipoprotein cholesterol; HR, hazard ratio; *N*, number of populations; Q, quartile; SBP, systolic blood pressure; SUA, serum uric acid; T2DM, type 2 diabetes mellitus; TG, triglycerides; WC, waist circumference.

* Quartiles were determined for the total population and separately for each gender.

** Per 1000 person‐years (95% CI).

**FIGURE 1:**
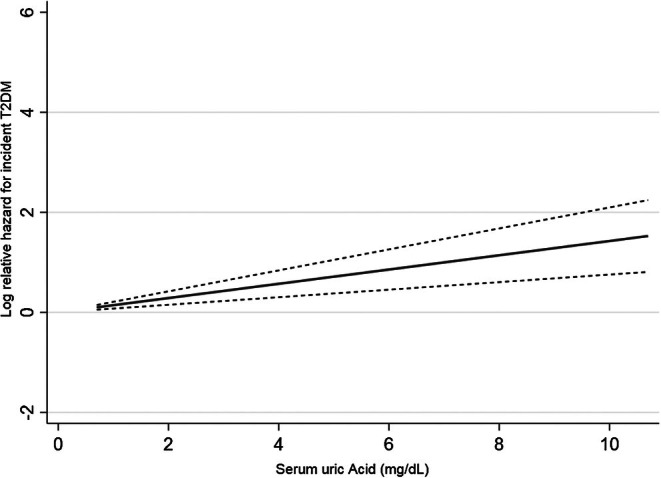
Dose–response relationship between SUA and risk of T2DM (95% confidence interval) Assessed by multivariable‐adjusted regression cubic spline model (*p* for trend < 0.001). SUA, serum uric acid; T2DM, type 2 diabetes mellitus.

Assessing the dose–response relationship between SUA and the risk of T2DM using the multivariable‐adjusted regression cubic spline model showed a linear association between SUA level and the risk of incident T2DM (Figure 1).

The results of Cox proportional hazard regression analysis exploring the association between SUA and incident T2DM are shown in Table 2. In the full adjustment model, each 1‐mg/dL increase in SUA was associated with an increased risk of incident T2DM by 18% for the whole population, 19% for men, and 26% for women; the corresponding HR (95% CI) were 1.18 (1.11–1.26), 1.19 (1.09–1.31), and 1.26 (1.14–1.38), respectively. Considering quartiles of SUA, a rising risk of incident T2DM is present across quartiles in the whole population (1.28 [1.00–1.63] for the second quartile, 1.57 [1.23–2.02] for the third quartile, and 1.82 [1.39–2.37] for the fourth quartile), *p* for trend < 0.001. Similarly, in the sex‐stratified analyses, we found increasing values of SUA were accompanied by the development of T2DM (both *p*s for trend ≤ 0.002). Furthermore, in women as well as the whole population, significant risks were found even in the second quartile (1.31 [1.01–1.72], and 1.28 [1.01–1.63], respectively). However, among men, this risk was significant only in the fourth quartile (2.47 [1.20–5.06]).

**FIGURE 2:**
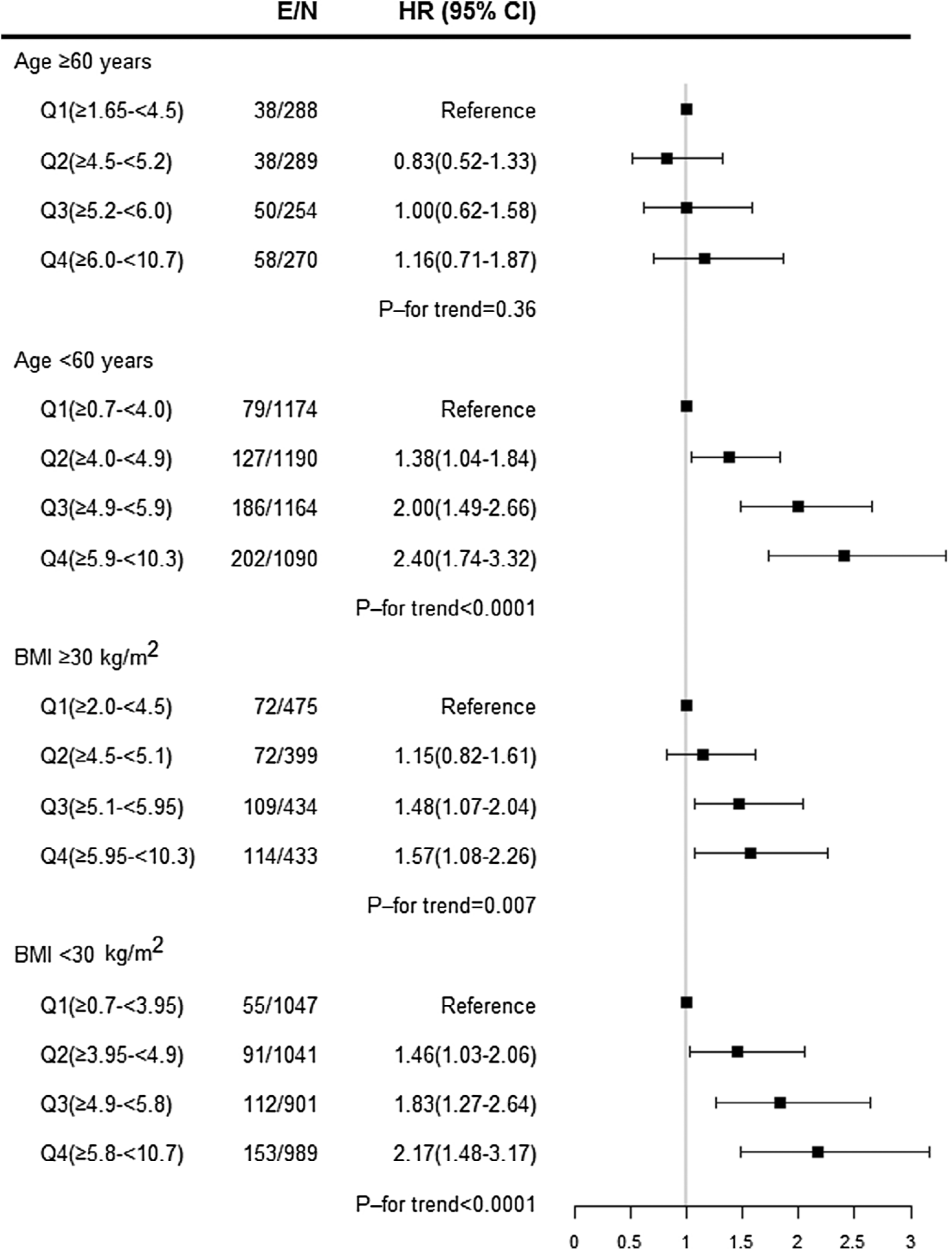
Association between SUA and the risk of T2DM by age groups and obesity status. BMI, body mass index; CI, confidence interval; HR, hazard ratio; Q, quartile; SUA, serum uric acid; T2DM, type 2 diabetes mellitus.

In subgroup analysis, we did not find a significant interaction between obesity status as well as age categories with SUA for incident T2DM, although the relationship between SUA and incident T2DM was more prominent among the younger population (Figure 2).

As shown in Table 3, our analysis revealed that adding FPG into the noninvasive ADA model resulted in a 10% increase in C‐index (95% CI: 76.2% [75.4–76.9]) and a 6.0% improvement in IDI (95% CI: 6% [4.2–7.6]) significantly. Although SUA was prominently associated with incident T2DM, by adding this biomarker to the ADA model, compared to its noninvasive model, the C‐index increased by only 1% (95% CI: 67.1% [65.5–68.7]) versus 66.2% (65.0–67.4). This suggests that incorporating SUA does not enhance the sensitivity of detecting individuals at risk for incident T2DM beyond the ADA noninvasive model alone. Moreover, a 1‐mg/dL increase in SUA was accompanied by HRs of 1.25, 1.17, 1.20, and 1.21 in models of ARIC without lipid measures, FOS, ARIC with lipid measures, and Iran's risk score, respectively; however, this approach did not improve the prediction of T2DM in terms of C‐index and IDI.

**TABLE 3:** Clinical performance of the different diabetes risk scores for incident T2DM by adding SUA.

	Risk score HR (95% CI)	FPG/SUA (mg/dL) HR (95% CI)	C‐index% (95% CI)	IDI % (95% CI)
ADA score (non‐invasive model)				
Model 1	1.72 (1.61 to 1.83)	—	66.2 (65.0 to 67.4)	
Model 2A	1.38 (1.27 to 1.47)	4.73 (4.15 to 5.39)	76.2 (75.4 to 76.9)	6.0 (4.2 to 7.6)
Model 2B	1.62 (1.50 to 1.73)	1.17 (1.10 to 1.23)	67.1 (65.5 to 68.7)	0.3 (−0.02 to 0.7)
ARIC score without lipid measures				
Model 1	2.12 (1.96 to 2.30)	—	73.3 (68.9 to 77.6)	
Model 2	2.08 (1.91 to 2.25)	1.25 (1.16 to 1.34)	74.5 (72.5 to 76.4)	0.9 (−0.05 to 1.8)
FOS score with lipid measures (HDL‐C only)				
Model 1	1.68 (1.59 to 1.77)		74.3 (72.8 to 75.7)	
Model 2	1.65 (1.56 to 1.74)	1.17 (1.09 to 1.26)	75.0 (71.5 to 78.4)	0.4 (−0.3 to 1.0)
ARIC score with lipid measures (HDL‐C and TG)				
Model 1	2.09 (1.93 to 2.26)		73.9 (72.0 to 75.6)	
Model 2	2.03 (1.88 to 2.20)	1.20 (1.12 to 1.29)	74.7 (72.6 to 76.8)	0.5 (−0.2 to 1.3)
Iran's score with lipid measures (HDL‐C and TG)				
Model 1	2.07 (1.92 to 2.32)		73.0 (69.4 to 77.3)	
Model 2	2.01 (1.86 to 2.17)	1.21 (1.12 to 1.30)	74.3 (70.3 to 78.3)	0.6 (−0.2 to 1.4)

*Note:* ADA score: included age groups, sex, parental history of diabetes, history of hypertension, high physical activity, and obesity status. Model 1: risk score without FPG. Model 2A: risk score + FPG alone. Model 2B: risk score + SUA alone. ARIC score: included age, race, FPG, SBP, WC, height, parental history of diabetes, and (HDL‐C, and TG in enhanced score). FOS: included age, sex, and parental history of diabetes, BMI, SBP, HDL‐C, WC, and FPG. Iran's score: included family history of diabetes, waist/height ratio, SBP, FPG, and TG/HDL‐C ratio. Model 1: risk score. Model 2: risk score + SUA.

Abbreviations: ADA, American Diabetes Association; ARIC, Atherosclerosis Risk in Communities; BMI, body mass index; CI, confidence interval; C‐index, concordance index; FOS, Framingham Offspring Study; FPG, fasting plasma glucose; HDL‐C, high‐density lipoprotein cholesterol; HR, hazard ratio; IDI, integrated discrimination improvement; SBP, systolic blood pressure; SUA, serum uric acid; T2DM, type 2 diabetes mellitus; TG, triglycerides; WC, waist circumference.

Among a subpopulation with insulin data (*n* = 3694), after adjustment for confounding factors as well as HOMA‐IR (instead of FPG), each 1‐mg/dL increment in SUA, increased the risk of incident T2DM by 15%, 13% and 21% in the whole population, men and women, respectively. Considering quartiles of SUA, overall rising risks of incident T2DM were observed across quartiles in the whole population, and both sexes (all *p*s for trend < 0.05) (Supplementary Table 3). Also, in participants with nutritional data (*n* = 3545), further adjustment for energy intake, red meat, organ meat, dairy products, and SSBs, a significant association between SUA (whether as a continuous or categorical variable) and incident T2DM was still found (Supplementary Table 4).

## 4 Discussion

In this population‐based study of Iranian urban residents during about a one‐decade follow‐up, we found a significant linear association between SUA and incident T2DM. After adjustment for a large set of confounders, we found that each 1‐mg/dL increment in SUA increased the risk of incident T2DM by 18%, 19%, and 26% for the whole population, men, and women, respectively; the relationship was not affected by sex, age categories, and obesity status. Moreover, even the second quartile of SUA (≥ 4.05 to < 5.00 mg/dL) for the whole population was accompanied by about 30% higher risk for T2DM. The association between SUA and incident T2DM persisted after further adjustment for insulin resistance and nutritional confounding factors. Importantly, despite the significant association between SUA and incident T2DM in the different well‐known predictive models of diabetes, its addition did not significantly improve the prediction of T2DM.

In a meta‐analysis, Xu et al. reported each 1‐mg/dL increase in SUA led to a 13.1% (95% CI: 8.4%–17.9%) increase in the risk of T2DM with significant heterogeneity among studies [8]. Recent cohort studies conducted in China [32] and Korea [33] reported that each 1‐mg/dL increase in SUA was also associated with a 9% and 13.8% increased risk of T2DM, respectively. The different results of studies that examined the association between SUA and T2DM might be explained by important factors such as study design (cross‐sectional versus prospective), sample size, age range of study population, definition of outcome, level of adjustment for confounders, and duration of follow‐up, as well as whether the study examined the whole population or each gender, separately.

In the current study applying stratified analysis, we found that the relationship between SUA and incident T2DM was consistent among men versus women, younger versus older, and obese versus non‐obese individuals. Similarly, in the meta‐analysis of Xu et al. no effect modification was found for age and sex [8]. In a prospective observation in Taiwan, Wu et al. demonstrated a positive gradient for the risk of T2DM across quartile of SUA in both genders and among those higher or lower than age 50 years [34]. In the Rotterdam study in the Netherlands SUA was associated with 23% incident T2DM among pre‐diabetic men but not women [35]. In line with our findings, a short‐term recent study among the Chinese population demonstrated a consistent positive association between SUA and incident T2DM among younger versus older, obese versus non‐obese as well as men versus women, although the association for women did not reach a significant level [32]. However, another cohort study in China [36] and a Panasonic cohort in Japan [37] demonstrated a significant positive relationship between SUA and incident T2DM only among women but not among men. Moreover, in the Korean retrospective cohort, hyperuricemia was a significant independent predictor of new‐onset T2DM up to 5‐year follow‐up in women, old age (≥ 55 years) and those with mild obesity (BMI: 24–30 kg/m^2^). However, the researchers did not show the level of interaction in their subgroup analysis [33].

In this study, we investigated the potential role of SUA in improving the prediction of T2DM. Our data analysis demonstrated that, despite the strong and independent association between SUA and incident T2DM, the addition of SUA did not improve the prediction of T2DM, even in the non‐invasive ADA prediction model which included non‐laboratory parameters. In general, an effect size greater than 0.8 for a predictor (i.e., HR ≥ 2) is commonly regarded as indicating a strong association, and incorporating such a variable into a model may lead to a meaningful improvement in the IDI index [38, 39]. Therefore, the HR of 1.18 observed in our multivariate analysis for each 1‐mg/dL increase in SUA level may not provide sufficient predictive strength to meaningfully enhance the discrimination of T2DM risk in term of both the C‐index and IDI across different prediction models. Moreover the proposed biological pathways indicate that SUA may influence inflammation and oxidative stress, which are linked to the development of various metabolic conditions. However, these pathways may not fully account for the complexities of disease progression or individual risk factors, thereby limiting SUA's predictive capacity in clinical models. A few studies examined the potential role of SUA in improving the prediction of T2DM. In Korean adults, the addition of SUA to the conventional model significantly improved the C‐index (0.66 versus 0.74) [40]. However, in a nested‐case control study among Swedish elderly women, smoking and serum adiponectin but not SUA significantly improved the prediction ability for T2DM [41]. Moreover, no Mendelian randomization study or randomized control trial has explored the causal role of SUA in the development of T2DM [42, 43].

Even though the mechanism of an increased risk of T2DM associated with high levels of SUA is poorly understood, several mechanisms may be proposed; SUA directly inhibits the insulin signaling pathway at the receptor level [44]. High SUA decreases glucose‐stimulated insulin secretion (GSIS) through the knockdown of human glucose transporter 9 (GLUT9), which is a urate transporter and also participates in pancreatic β‐cell function [45]. In addition, SUA increases oxidative stress, inflammation, and endothelial dysfunction that is accompanied by impairment in nitric oxide (NO) production leading to vasoconstriction, insulin resistance, and impaired glucose uptake in the muscles [45, 46]. On the other hand, inducible NO synthase (iNOS)‐derived NO production causes a decrease in GSIS and β‐cell apoptosis [45].

The strengths of our study included a population‐based prospective design with a large sample size and long‐term follow‐up, adjustment for a large set of confounders including nutritional data and HOMA‐IR, and assessment of the added value of SUA to the different prediction models for T2DM. However, there were some limitations; the effect of residual confounders such as alcohol consumption, uric acid lowering drugs, and particularly history of gout (as a strong confounder) was not entered in our data analysis since no data regarding these variables were acquired in the TLGS questionnaire. Our study is limited to the urban residents of the metropolitan city of Tehran, so our findings might not extrapolate to the rural zone of the country.

## 5 Conclusions

In conclusion, we observed a significant linear association between SUA and incident T2DM. Despite this, adding SUA to different prediction models did not provide better risk prediction for T2DM.

## Author Contributions

F.A., F.H., and M.T. were responsible for the conceptualization and design of the study. F.H., M.T., S.A., and N.K. scheduled the methodological aspects of the study. M.T. planned, supervised, and validated the laboratory analyses. S.A. and S.H.‐N. conducted the statistical analyses. F.H., M.T., N.K., and S.A. contributed to the interpretation of the data. S.H.‐N. managed, analyzed, and interpreted the nutritional data. N.K. and M.T. drafted the initial version of the manuscript. F.A., F.H., M.T., and N.K. critically reviewed and revised the manuscript. All authors have read, reviewed, and approved the final manuscript.

## Funding

The authors have nothing to report.

## Ethics Statement

The Institutional Review Board of the Research Institute for Endocrine Sciences, Shahid Beheshti University of Medical Sciences, Tehran, Iran, approved this study (IR.SBMU.ENDOCRINE.REC.1403.107).

## Consent

From all participants, a written informed consent was obtained.

## Conflicts of Interest

The authors declare no conflicts of interest.

## Supporting information


**Supplementary Table 1.** Baseline characteristics of the men by SUA quartiles: Tehran Lipid and Glucose Study (2009–2012).

**Supplementary Table 2.** Baseline characteristics of the women by SUA quartiles: Tehran Lipid and Glucose Study (2009–2012).

**Supplementary Table 3.** Incident T2DM by quartiles and 1 mg/dL increase of SUA among subpopulation with insulin data.

**Supplementary Table 4.** Incident T2DM by quartiles and 1 mg/dL increase of SUA among subpopulation with nutritional data.

## Data Availability

The data that support the findings of this study are available on request from the corresponding author. The data are not publicly available due to privacy or ethical restrictions.
