# Supplementary material for: The Role of Serum Uric Acid in the Prediction of Type 2 Diabetes Mellitus: Tehran Lipid and Glucose Study
Source: J Clin Lab Anal. 2026 Jul 24;40(17):e70314. doi: 10.1002/jcla.70314 (PMC13400966; doi:10.1002/jcla.70314)
Supplement: Supplementary file 2 [file JCLA-40-e70314-s002.docx]

| Supplementary Table 1. Baseline characteristics of the men by SUA quartiles: Tehran Lipid and Glucose Study (2009-2012) | | | | | | | | |
| --- | --- | --- | --- | --- | --- | --- | --- | --- |
|  |  |  |  | **SUA Quartiles (mg/dL)** | | | | |
| Variables^*^ |  | **Whole population**  **n=2588** |  | **Q1 (≥2.15-<4.05)**  **n = 147** | **Q2 (≥4.05-<5.00)**  **n = 481** | **Q3 (≥5.00-<5.90)**  **n =810** | **Q4 (≥ 5.90-<10.30)**  **n =1150** | ***P* value^**^** |
| Age (years) |  | 48.54 ± 12.80 |  | 50.16 ± 12.98 | 49.30 ± 12.86 | 49.32 ± 12.94 | 47.46 ± 12.56 | 0.001 |
| WC (cm) |  | 96.68 ± 10.44 |  | 90.04 ± 10.30 | 92.39 ± 10.80 | 96.54 ± 10.17 | 99.41 ± 9.46 | < 0.001 |
| BMI (kg/m^2^) |  | 27.13 ± 4.07 |  | 24.48 ± 3.61 | 25.58 ± 4.05 | 26.94 ± 3.90 | 28.24 ± 3.86 | < 0.001 |
| SBP (mmHg) |  | 119.07 ± 16.36 |  | 115.10 ± 16.11 | 116.56 ± 15.39 | 118.72 ± 16.25 | 120.87 ± 16.64 | < 0.001 |
| DBP (mmHg) |  | 79.83 ± 10.51 |  | 76.70 ± 9.74 | 77.99 ± 9.59 | 79.34 ± 10.32 | 81.34 ± 10.87 | < 0.001 |
| FPG (mg/dL) |  | 95.33 ± 8.86 |  | 92.72 ± 7.07 | 94.58 ± 8.70 | 95.75 ± 9.04 | 95.68 ± 8.93 | < 0.001 |
| TG (mg/dL) |  | 141 (100-198) |  | 106 (78-144) | 117 (84-155) | 134 (99-180) | 164 (118-228) | < 0.001 |
| HDL-C (mg/dL) |  | 42.36 ± 9.20 |  | 45.90 ± 10.38 | 44.10 ± 9.27 | 43.22 ± 9.04 | 40.57 ± 8.78 | < 0.001 |
| SUA (mg/dL) |  | 5.77 ± 1.15 |  | 3.65 ± 0.34 | 4.58 ± 0.25 | 5.42 ± 0.25 | 6.78 ± 0.78 | < 0.001 |
| eGFR (mL/min/1.73m^2^) |  | 84.35 ± 13.00 |  | 88.36 ± 14.55 | 87.51 ± 12.20 | 84.87 ± 12.00 | 82.15 ± 13.38 | < 0.001 |
| Current smoker (yes, %) |  | 860 (33.23) |  | 65 (44.22) | 161 (33.47) | 252 (31.11) | 382 (33.22) | 0.022 |
| Low physical activity (yes, %) |  | 1125 (43.47) |  | 61 (41.50) | 197 (40.96) | 396 (45.56) | 498 (43.30) | 0.405 |
| Education |  |  |  |  |  |  |  | < 0.001 |
| < 6 years |  | 424 (16.38) |  | 31 (21.09) | 96 (19.96) | 139 (17.16) | 158 (13.74) |  |
| 6-12 years |  | 1,436 (55.49) |  | 87 (59.18) | 268 (55.72) | 468 (57.78) | 613 (53.30) |  |
| > 12 years |  | 728 (28.13) |  | 29 (19.73) | 117 (24.32) | 203 (25.06) | 379 (32.96) |  |
| History of CVD (yes, %) |  | 218 (8.42) |  | 11 (7.48) | 41 (8.52) | 79 (9.75) | 87 (7.57) | 0.372 |
| FH-DM (yes, %) |  | 194 (7.50) |  | 10 (6.80) | 24 (4.99) | 59 (7.28) | 101 (8.78) | 0.064 |
| Lipid-lowering medications (yes, %) |  | 151 (5.83) |  | 5 (3.40) | 30 (6.24) | 43 (5.31) | 73 (6.35) | 0.443 |
| Antihypertensive medications (yes, %) |  | 209 (8.08) |  | 8 (5.44) | 31 (6.44) | 66 (8.15) | 104 (9.04) | 0.208 |
| SUA, serum uric acid; Q, quartile; n, number; WC, waist circumference; BMI, body mass index; SBP, systolic blood pressure; DBP, diastolic blood pressure; FPG, fasting plasma glucose; TG, triglycerides; HDL-C, high-density lipoprotein cholesterol; eGFR, estimated glomerular filtration rate; CVD, cardiovascular diseases; FH-DM, family history of type 2 diabetes mellitus; SD, standard deviation; IQR, interquartile range  * Data are presented as mean ± SD for normally distributed continuous variables, median (IQR) for triglycerides (TG) with a skewed distribution, and number (%) for categorical variables.  ** *P* values correspond to the ANOVA test for normally distributed continuous variables, the Chi-squared test for categorical variables, and the Kruskal–Wallis test for skewed or ordinal variables. | | | | | | | | |
