# Supplementary material for: The Role of Serum Uric Acid in the Prediction of Type 2 Diabetes Mellitus: Tehran Lipid and Glucose Study
Source: J Clin Lab Anal. 2026 Jul 24:e70314. Online ahead of print. doi: 10.1002/jcla.70314 (PMC13400966; doi:10.1002/jcla.70314)
Supplement: Supplementary file 2 — Supplementary Table 2. Baseline characteristics of the women by SUA quartiles: Tehran Lipid and Glucose Study (2009–2012). [file JCLA-9999-e70314-s002.docx]

| Supplementary Table 2. Baseline characteristics of the women by SUA quartiles: Tehran Lipid and Glucose Study (2009-2012) | | | | | | | | |
| --- | --- | --- | --- | --- | --- | --- | --- | --- |
|  |  |  |  | **SUA Quartiles (mg/dL)** | | | | |
| Variables^*^ |  | **Whole population**  **n = 3131** |  | **Q1 (≥0.70-<4.10)**  **n = 1350** | **Q2 (≥4.10-<5.00)**  **n = 1030** | **Q3 (≥5.00-<5.85)**  **n = 506** | **Q4 (≥ 5.85-≤10.70)**  **n = 245** | ***P* value^**^** |
| Age (year) |  | 47.60 ± 12.10 |  | 44.59 ± 10.92 | 48.56 ± 12.18 | 49.80 ± 12.37 | 55.52 ± 12.19 | < 0.001 |
| WC (cm) |  | 93.89 ± 11.67 |  | 89.80 ± 10.62 | 94.95 ± 10.88 | 98.53 ± 11.34 | 102.41 ± 12.04 | < 0.001 |
| BMI (kg/m^2^) |  | 29.32 ± 11.11 |  | 27.47 ± 4.30 | 29.55 ± 4.63 | 32.23 ± 25.20 | 32.48 ± 5.43 | < 0.001 |
| SBP (mmHg) |  | 114.59 ± 17.85 |  | 110.28 ± 15.89 | 115.39 ± 17.55 | 119.23 ± 19.46 | 125.43 ± 18.43 | < 0.001 |
| DBP (mmHg) |  | 75.92 ± 10.63 |  | 73.61 ± 10.06 | 76.64 ± 10.49 | 78.66 ± 10.78 | 80.00 ± 11.00 | < 0.001 |
| FPG (mg/dL) |  | 93.49 ± 9.00 |  | 91.82 ± 8.30 | 93.72 ± 8.79 | 95.37 ± 9.83 | 97.84 ± 9.58 | < 0.001 |
| TG (mg/dL) |  | 116 (83-164) |  | 97 (71-131) | 125 (90-175) | 142 (103-191) | 167 (124-229) | < 0.001 |
| HDL-C (mg/dL) |  | 51.32 ± 11.34 |  | 53.01 ± 11.24 | 51.04 ± 11.47 | 49.36 ± 11.04 | 47.16 ± 10.11 | < 0.001 |
| SUA (mg/dl) |  | 4.31 ± 1.08 |  | 3.38 ± 0.48 | 4.48 ± 0.26 | 5.32 ± 0.25 | 6.67 ± 0.80 | < 0.001 |
| eGFR (mL/min/1.73m^2^) |  | 79.71 ± 13.58 |  | 84.26 ± 12.36 | 78.82 ± 12.66 | 75.57 ± 13.05 | 66.96 ± 13.32 | < 0.001 |
| Current smoker (yes, %) |  | 217 (6.93) |  | 90 (6.67) | 76 (7.38) | 41 (8.10) | 10 (4.08) | 0.201 |
| Low physical activity (yes, %) |  | 1138 (36.35) |  | 511 (37.85) | 355 (34.47) | 172 (33.99) | 100 (40.82) | 0.101 |
| Education |  |  |  |  |  |  |  | < 0.001 |
| < 6 years |  | 862 (27.53) |  | 277 (20.52) | 308 (29.90) | 165 (32.61) | 112 (45.71) |  |
| 6-12 years |  | 1,665 (53.18) |  | 753 (55.78) | 537 (52.14) | 262 (51.78) | 113 (46.12) |  |
| > 12 years |  | 604 (19.29) |  | 320 (23.70) | 185 (17.96) | 79 (15.61) | 20 (8.16) |  |
| History of CVD (yes, %) |  | 177 (5.65) |  | 41 (3.04) | 64 (6.21) | 37 (7.31) | 35 (14.29) | < 0.001 |
| FH-DM (yes, %) |  | 354 (11.31) |  | 149 (11.04) | 126 (12.23) | 55 (10.87) | 24 (9.80) | 0.652 |
| Lipid-lowering medications (yes, %) |  | 230 (7.35) |  | 51 (3.78) | 90 (8.74) | 56 (11.07) | 33 (13.47) | < 0.001 |
| Antihypertensive medications (yes, %) |  | 349 (11.15) |  | 76 (5.63) | 129 (12.52) | 75 (14.82) | 69 (28.16) | < 0.001 |
| Menopausal status (yes, %) |  | 1,236 (39.5) |  | 362 (26.8) | 450 (43.7) | 255 (40.4) | 169 (69.0) | < 0.001 |
| SUA, serum uric acid; Q, quartile; n, number; WC, waist circumference; BMI, body mass index; SBP, systolic blood pressure; DBP, diastolic blood pressure; FPG, fasting plasma glucose; TG, triglycerides; HDL-C, high-density lipoprotein cholesterol; eGFR, estimated glomerular filtration rate; CVD, cardiovascular diseases; FH-DM, family history of type 2 diabetes mellitus; SD, standard deviation; IQR, interquartile range  * Data are presented as mean ± SD for normally distributed continuous variables, median (IQR) for triglycerides (TG) with a skewed distribution, and number (%) for categorical variables.  ** *P* values correspond to the ANOVA test for normally distributed continuous variables, the Chi-squared test for categorical variables, and the Kruskal–Wallis test for skewed or ordinal variables. | | | | | | | | |
