# Supplementary material for: The Role of Serum Uric Acid in the Prediction of Type 2 Diabetes Mellitus: Tehran Lipid and Glucose Study
Source: J Clin Lab Anal. 2026 Jul 24:e70314. Online ahead of print. doi: 10.1002/jcla.70314 (PMC13400966; doi:10.1002/jcla.70314)
Supplement: Supplementary file 3 — Supplementary Table 3. Incident T2DM by quartiles and 1 mg/dL increase of SUA among subpopulation with insulin data. [file JCLA-9999-e70314-s003.docx]

| Supplementary Table 3. Incident T2DM by quartiles and 1 mg/dL increase of SUA among subpopulation with insulin data^*^ | | | | | | | | | |
| --- | --- | --- | --- | --- | --- | --- | --- | --- | --- |
|  | |  | **Quartiles of SUA (mg/dL)^**^** | | | | |  | **1 mg/dL**  **increase of SUA** |
| Whole population | |  | **Q1 (≥0.7-<4.05)** | **Q2 (≥4.05-<5.00)** | **Q3 (≥5.00-<5.85)** | **Q4 (≥ 5.85-<10.70)** | ***P* for trend** |  |  |
|  | **E/N** |  | 86/1067 | 125/972 | 146/839 | 157/816 |  |  | 514/3694 |
|  | **Model 1** |  | Reference | 1.60 (1.27-2.21) | 2.56 (1.92-3.40) | 3.18 (2.37-4.28) | < 0.001 |  | 1.37 (1.27-1.47) |
|  | **Model 2** |  | Reference | 1.35 (1.02-1.80) | 1.66 (1.24-2.24) | 1.66 (1.20-2.30) | 0.002 |  | 1.15 (1.06-1.25) |
|  |  |  |  |  |  |  |  |  |  |
| Men | |  | **Q1 (≥2.15-<4.05)** | **Q2 (≥4.05-<5.00)** | **Q3 (≥5.00-<5.90)** | **Q4(≥ 5.90-<10.30)** | ***P* for trend** |  |  |
|  | **E/N** |  | 5/102 | 31/289 | 80/518 | 118/660 |  |  | 234/1569 |
|  | **Model 1** |  | Reference | 2.46 (0.96-6.32) | 3.80 (1.54-9.37) | 4.71 (1.92-11.53) | < 0.001 |  | 1.31 (1.17-1.45) |
|  | **Model 2** |  | Reference | 2.06 (0.79-5.36) | 2.85 (1.14-7.14) | 2.74 (1.10-6.88) | 0.04 |  | 1.13 (1.00-1.28) |
|  |  |  |  |  |  |  |  |  |  |
| Women | |  | **Q1 (≥0.70-<4.10)** | **Q2 (≥4.10-<5.00)** | **Q3 (≥5.00-<5.85)** | **Q4 (≥ 5.85-<10.70)** | ***P* for trend** |  |  |
|  | **E/N** |  | 81/965 | 94/683 | 66/321 | 39/156 |  |  | 280/2125 |
|  | **Model 1** |  | Reference | 1.66 (1.23-2.24) | 2.52 (1.81-3.51) | 3.22 (2.17-4.78) | < 0.001 |  | 1.45 (1.32-1.60) |
|  | **Model 2** |  | Reference | 1.35 (0.99-1.84) | 1.58 (1.11-2.26) | 1.65 (1.06-2.57) | 0.01 |  | 1.21 (1.07-1.37) |
| T2DM, type 2 diabetes mellitus; SUA, serum uric acid; Q, quartile; E: number of events, N: number of populations; BMI, body mass index; WC, waist circumference; CVD, cardiovascular diseases; FH-DM, family history of type 2 diabetes mellitus; SBP, systolic blood pressure; TG, triglycerides; HDL-C, high-density lipoprotein cholesterol; eGFR, estimated glomerular filtration rate; HOMA-IR, homeostasis model assessment for insulin resistance.  Model 1: adjusted for age and sex in the whole population, and just for age in the sex-stratified analyses.  Model 2: Model 1 + adjusted for BMI, WC, education, current smoker, low physical activity, history of CVD, FH-DM, anti-hypertensive medications, lipid-lowering medication, SBP, TG/HDL-C, eGFR and HOMA-IR (+ menopausal status among women).  Data presented as hazard ratio (95% confidence interval)  * Number of subjects with insulin data: 3694  ** Quartiles were determined for the total population and separately for each gender. | | | | | | | | | |
