# Supplementary material for: The Role of Serum Uric Acid in the Prediction of Type 2 Diabetes Mellitus: Tehran Lipid and Glucose Study
Source: J Clin Lab Anal. 2026 Jul 24:e70314. Online ahead of print. doi: 10.1002/jcla.70314 (PMC13400966; doi:10.1002/jcla.70314)
Supplement: Supplementary file 4 — Supplementary Table 4. Incident T2DM by quartiles and 1 mg/dL increase of SUA among subpopulation with nutritional data. [file JCLA-9999-e70314-s001.docx]

| Supplementary Table 4. Incident T2DM by quartiles and 1 mg/dL increase of SUA among subpopulation with nutritional data^*^ | | | | | | | | | |
| --- | --- | --- | --- | --- | --- | --- | --- | --- | --- |
|  | |  | **Quartiles of SUA (mg/dL)^**^** | | | | |  | **1 mg/dL**  **increase of SUA** |
| Whole population | |  | **Q1 (≥0.70-<4.05)** | **Q2 (≥4.05-<5.00)** | **Q3 (≥5.00-<5.90)** | **Q4 (≥ 5.90-<10.30)** | ***P* for trend** |  |  |
|  | **E/N** |  | 73/975 | 103/923 | 128/797 | 166/850 |  |  | - |
|  | **Model 1** |  | Reference | 1.19 (0.89-1.62) | 1.62 (1.17-2.37) | 1.93 (1.37-2.73) | <0.001 |  | 1.20 (1.10-1.31) |
|  | **Model 2** |  | Reference | 1.20 (0.88-1.64) | 1.59 (1.16-2.20) | 1.92 (1.36-2.71) | <0.001 |  | 1.20 (1.10-1.30) |
|  | **Model 3** |  | Reference | 1.20 (0.88-1.64) | 1.59 (1.16-2.20) | 1.92 (1.36-2.71) | <0.001 |  | 1.20 (1.10-1.30) |
|  | **Model 4** |  | Reference | 1.20 (0.88-1.63) | 1.58 (1.14-2.18) | 1.91 (1.35-2.69) | <0.001 |  | 1.19 (1.10-1.30) |
| Men | |  | **Q1 (≥2.15-<4.05)** | **Q2 (≥4.05-<5.00)** | **Q3 (≥5.00-<5.90)** | **Q4(≥ 5.90-<10.30)** | ***P* for trend** |  |  |
|  | **E/N** |  | 4/96 | 30/295 | 68/509 | 131/728 |  |  | - |
|  | **Model 1** |  | Reference | 1.85 (0.65-5.30) | 2.05 (0.74-5.68) | 2.87 (1.05-7.89) | 0.002 |  | 1.23 (1.09-1.39) |
|  | **Model 2** |  | Reference | 1.89 (0.66-5.42) | 2.06 (0.75-5.71) | 2.94 (1.07-8.09) | 0.002 |  | 1.22 (1.08-1.38) |
|  | **Model 3** |  | Reference | 1.89 (0.66-5.42) | 2.07 (0.75-5.71) | 2.94 (1.07-8.09) | 0.002 |  | 1.22 (1.08-1.38) |
|  | **Model 4** |  | Reference | 1.85 (0.64-5.30) | 2.01 (0.73-5.58) | 2.87 (1.04-7.88) | 0.003 |  | 1.22 (1.08-1.37) |
| Women | |  | **Q1 (≥0.70-<4.10)** | **Q2 (≥4.10-<5.00)** | **Q3 (≥5.00-<5.90)** | **Q4 (≥ 5.90-<9.00)** | ***P* for trend** |  |  |
|  | **E/N** |  | 69/879 | 73/628 | 60/288 | 35/122 |  |  | - |
|  | **Model 1** |  | Reference | 1.19 (0.84-1.68) | 1.76 (1.19-2.59) | 1.96 (1.22-3.15) | 0.001 |  | 1.26 (1.11-1.44) |
|  | **Model 2** |  | Reference | 1.19 (0.84-1.69) | 1.73 (1.17-2.56) | 1.96 (1.22-3.15) | 0.001 |  | 1.27 (1.12-1.44) |
|  | **Model 3** |  | Reference | 1.19 (0.84-1.69) | 1.73 (1.17-2.56) | 1.97 (1.23-3.18) | 0.001 |  | 1.27 (1.12-1.44) |
|  | **Model 4** |  | Reference | 1.18 (0.83-1.68) | 1.71 (1.16-2.53) | 1.96 (1.22-3.14) | 0.001 |  | 1.27 (1.11-1.44) |
| T2DM, type 2 diabetes mellitus; SUA, serum uric acid; Q, quartile; E: number of events, N: number of populations; BMI, body mass index; WC, waist circumference; CVD, cardiovascular diseases; FH-DM, family history of type 2 diabetes mellitus; SBP, systolic blood pressure; TG, triglycerides; HDL-C, high-density lipoprotein cholesterol; eGFR, estimated glomerular filtration rate; FPG, fasting plasma glucose; SSBs, sugar-sweetened beverages.  Model 1: adjusted for age, sex (only for the whole population), BMI, WC, education, current smoker, low physical activity, history of CVD, FH-DM, anti-hypertensive medications, lipid-lowering medication, SBP, TG/HDL-C, eGFR, and FPG (+ menopausal status among women).  Model 2: Model 1 + total energy intake + red meat and organ meat.  Model 3: Model 2 + dairy products.  Model 4: Model 2 + SSBs.  Data presented as hazard ratio (95% confidence interval)  * Number of subjects with nutritional data: 3545  ** Quartiles were determined for the total population and separately for each gender. | | | | | | | | | |
